# Does the low prevalence affect the sample size of interventional clinical trials of rare diseases? An analysis of data from the aggregate analysis of clinicaltrials.gov

**DOI:** 10.1186/s13023-017-0597-1

**Published:** 2017-03-02

**Authors:** Siew Wan Hee, Adrian Willis, Catrin Tudur Smith, Simon Day, Frank Miller, Jason Madan, Martin Posch, Sarah Zohar, Nigel Stallard

**Affiliations:** 1 Statistics and Epidemiology Unit, Division of Health Sciences, Warwick Medical School, University of Warwick, Coventry, CV4 7AL UK; 2 Warwick Clinical Trials Unit, Division of Health Sciences, Warwick Medical School, University of Warwick, Coventry, CV4 7AL UK; 3 North West Hub for Trials Methodology Research, Department of Biostatistics, Liverpool, L69 3GL UK; 4 Clinical Trials Consulting and Training Limited, Buckingham, UK; 5 Department of Statistics, Stockholm University, Stockholm, Sweden; 6 Section of Medical Statistics, CeMSIIS, Medical University of Vienna, Vienna, Austria; 7 INSERM, U1138, team 22, Centre de Recherche des Cordeliers, Université Paris 5, Université Paris 6, Paris, France

**Keywords:** Aggregate analysis of clinialtrials.gov, Orphadata, Orphanet, Prevalence, Orphan disease, Rare disease, Sample size

## Abstract

**Background:**

Clinical trials are typically designed using the classical frequentist framework to constrain type I and II error rates. Sample sizes required in such designs typically range from hundreds to thousands of patients which can be challenging for rare diseases. It has been shown that rare disease trials have smaller sample sizes than non-rare disease trials. Indeed some orphan drugs were approved by the European Medicines Agency based on studies with as few as 12 patients. However, some studies supporting marketing authorisation included several hundred patients. In this work, we explore the relationship between disease prevalence and other factors and the size of interventional phase 2 and 3 rare disease trials conducted in the US and/or EU. We downloaded all clinical trials from Aggregate Analysis of ClinialTrials.gov (AACT) and identified rare disease trials by cross-referencing MeSH terms in AACT with the list from Orphadata. We examined the effects of prevalence and phase of study in a multiple linear regression model adjusting for other statistically significant trial characteristics.

**Results:**

Of 186941 ClinicalTrials.gov trials only 1567 (0.8%) studied a single rare condition with prevalence information from Orphadata. There were 19 (1.2%) trials studying disease with prevalence <1/1,000,000, 126 (8.0%) trials with 1–9/1,000,000, 791 (50.5%) trials with 1–9/100,000 and 631 (40.3%) trials with 1–5/10,000. Of the 1567 trials, 1160 (74%) were phase 2 trials. The fitted mean sample size for the rarest disease (prevalence <1/1,000,000) in phase 2 trials was the lowest (mean, 15.7; 95% CI, 8.7–28.1) but were similar across all the other prevalence classes; mean, 26.2 (16.1–42.6), 33.8 (22.1–51.7) and 35.6 (23.3–54.3) for prevalence 1–9/1,000,000, 1–9/100,000 and 1–5/10,000, respectively. Fitted mean size of phase 3 trials of rarer diseases, <1/1,000,000 (19.2, 6.9–53.2) and 1–9/1,000,000 (33.1, 18.6–58.9), were similar to those in phase 2 but were statistically significant lower than the slightly less rare diseases, 1–9/100,000 (75.3, 48.2–117.6) and 1-5/10,000 (77.7, 49.6–121.8), trials.

**Conclusions:**

We found that prevalence was associated with the size of phase 3 trials with trials of rarer diseases noticeably smaller than the less rare diseases trials where phase 3 rarer disease (prevalence <1/100,000) trials were more similar in size to those for phase 2 but were larger than those for phase 2 in the less rare disease (prevalence ≥1/100,000) trials.

## Background

The European Union (EU) define a disease as being rare if the prevalence is not more than 5 in 10,000 which affects approximately 254,500 people throughout the EU member countries whose total population is approximately 509 million [1]. The United States (US) define a disease as being rare if it affects fewer than 200,000 person in the US [2]. This is equivalent to 62 people in 100,000 in 2015 [1]. In such circumstances, one may still be able to design a randomised controlled trial (RCT) based on the classical frequentist framework where, for example, the sample size for a two sample *t*-test with a 0.05 two-sided type I error rate and 0.90 power to detect a standardized effect size of 0.20 is 1052.

As stated in the “Guideline on clinical trials in small populations” by the European Medicines Agency/Committee for Medicinal Products for Human Use (EMA/CHMP), most orphan indications submitted for regulatory approval are based on RCTs [3]. Deviation from the perceived gold standard RCT is uncommon. This statement is supported by Buckley [4], who presented a short summary of clinical trials of drugs for rare diseases approved by the European regulator between 2001 and 2007. Some of these studies had as few as 12 patients and some several hundreds. For example, the marketing authorisation of carglumic acid for hyperammonaemia due to N-acetyl glutamate synthase deficiency was supported by one pharmacokinetic study with 12 patients and one retrospective study with 20 patients. In contrast, the marketing authorisation of sorafenib tosilate for renal cell and hepatocellular carcinomas was supported by one phase III renal trial with 903 patients and one phase III hepatic trial with 602 patients.

Bell and Tudur Smith compared the characteristics of rare and non-rare disease clinical trials registered in ClinicalTrials.gov [5]. In their review, 64% of rare disease trials had fewer than 50 patients compared to 38% of non-rare disease trials. Only 14% of rare disease trials had more than 100 patients compared to 36% of non-rare disease trials. These results suggest that large studies are possible when studying indications for rare diseases. However, many rare diseases affect 1 in 100,000 or fewer [6] limiting the potential pool of patients that would be eligible and willing to be recruited to trials. Accordingly, the design and analysis of clinical trials for these diseases becomes more challenging. In addition, as stated in the EMA/CHMP guideline, the prevalence of the disease may constrain to varying degrees the design, conduct, analysis and interpretation of these trials.

In this paper we examine the association between the disease prevalence and sample size for clinical trials in rare diseases allowing for other factors, extending the work of Bell and Tudur Smith but without comparison between non-rare and rare disease trials. Our analysis is based on data from the Aggregate Analysis of ClinicalTrials.gov database (AACT) [7], a registry of more than 180,000 clinical studies and Orphadata [8], a portal for information of rare diseases and their prevalence.

## Methods

### Aggregate analysis of clinicaltrials.gov database (AACT)

The database from ClinicalTrials.gov, Aggregate Analysis of ClinicalTrials.gov (AACT), is comprised of clinical studies registered up to 27 September 2015 [7, 9]. A comprehensive documentation of definitions of all variables is available on the ClinicalTrials.gov Protocol Registration System [10]. Each study in AACT may have information on the study characteristics such as types of study (interventional, observational, patient registry, or expanded access), phase of investigation (phase 1, 2, 3 or 4), design features of the study such as the intervention model (crossover, factorial, parallel or single group assignment), masking (double blind, single blind or open label), allocation (randomized, non-randomized), primary endpoint (e.g., efficacy, safety, pharmacodynamics, pharmacokinetics), number of intervention arms, and lead sponsor (industry, National Institutes of Health (NIH), US Federal Agency or other). Also recorded is the date that enrolment began, primary completion date which is either the date when the final subject was examined or received an intervention for the purpose of data collection for the primary outcome or the anticipated date when this will occur, the actual sample size upon completion of the study or the anticipated sample size for trials that have not yet completed recruitment, recruitment status, whether or not the trial had a Data Monitoring Committee (DMC), whether or not the intervention was Food and Drug Administration (FDA) regulated, and, for a trial with an FDA-regulated intervention whether or not this was an “applicable clinical trial” as defined under Section 801 of FDA Amendments Act (FDAAA801). Briefly, an applicable clinical trial is one where the trial has one or more sites in the US, is conducted under an FDA investigational new drug application or the regulated intervention (drug, biological product or device) is manufactured in the US and is to be exported for research.

Other clinical characteristics available from the AACT include the inclusion/eligibility criteria such as gender (female, male or both), age range of participants and whether or not the trial accepts healthy volunteers. The primary conditions or diseases being studied were recorded using the National Library of Medicine’s (NLM) Medical Subject Headings (MeSH) when possible.

### Orphadata

Orphadata is a database of rare diseases compiled by the 40-country consortium Orphanet coordinated by the French National Institute of Health and Medical Research (INSERM) team [11]. It gives an inventory of rare diseases comprised of the typology of the disease and cross-referencing with external classifications such as the 10^th^ International Classification of Diseases (ICD-10), Online Mendelian Inheritance in Man (OMIM), United Medical Language System (UMLS), MeSH and Medical Dictionary for Regulatory Activities (MedDRa). Orphadata, also contains epidemiology data such as type of prevalence (point prevalence, birth prevalence, lifetime prevalence, incidence, or the number of cases/families, see Posada de la Paz et al. for definition of types of prevalence [12]) by geographical area (e.g., country, continent), average age of onset, clinical signs, and for some rare disorders, the associated genes and their influences in the pathogenesis of the disease [8]. Prevalence data for rare conditions in Orphadata are classified into six possible classes, namely, <1/1,000,000, 1–9/1,000,000, 1–9/100,000, 1–5/10,000, 6–9/10,000, >1/1,000, along with “not yet documented” and “unknown”. These data were obtained from either published literature or registries. Some information from published literature were yet to be validated by experts and so the entry was recorded as “Not yet validated”. For our work, we focus on the epidemiological data such as type and class of prevalence data and geographical area.

Figure 3 (see Appendix 1) shows that there were 9199 rare diseases in Orphadata but only 5029 (55%) had prevalence information. Of the 5029 diseases about one third (1585) of the entries had only one type of prevalence entry. The other 3444 had more than one type of prevalence entry giving a total of 10008 entries. A total of 8060 of these entries had been validated.

### Merging of AACT and Orphadata

AACT and Orphadata were downloaded on 9 May 2016. A technical description of the merging of AACT and Orphadata is given in Appendix 2. For our analysis, we identified trials in AACT by matching the MeSH terms in AACT with those in Orphadata. Trials in AACT with MeSH terms not in Orphadata were declared as trials not studying non-rare diseases. In our work, focus was restricted to interventional phase 2 and/or 3 trials in a single rare disease with treatment as the primary purpose conducted in the US only, EU only (member states of the EU and associated countries) or in both US and EU. We restricted trials to these countries only because we believe that rare disease prevalence was well estimated and homogeneous in these countries whereas the prevalence for some diseases varies very considerably between US/EU and some other countries. Trials studying more than one rare disease were excluded from further analyses because it was unclear which disease prevalence data should be used.

For each trial, the prevalence of the disease in the countries where the trial took place was identified. If there was more than one prevalence entry for the disease in the trial location, the prevalence was used based on the following variables (in decreasing order of preference):

1. Validation status: (i) validated, and (ii) not yet validated.
2. Type of prevalence: (i) point prevalence, (ii) lifetime prevalence, (iii) prevalence at birth, (iv) annual incidence, and (v) cases/families.

If no prevalence information was available for the disease in the trial location then the prevalence for a neighbouring country or another country from the same geographic region was used. See Appendix 2 for details on the merging of diseases and their class of prevalence. Note that if only the number of cases/families was recorded, the prevalence was assumed to be <1/1,000,000.

Figure 4 (see Appendix 3) shows that there were 186941 trials in ClinicalTrials.gov and of these 28547 were interventional phase 2 and/or 3 treatment trials conducted in the US and/or EU. There were 2136 trials that studied rare conditions only and of these 2019 studied one rare condition only. Of the 2019 trials of a single disease in the Orphadata database, 415 were excluded from analyses; 16 because they had prevalence greater than 5/10,000 (because a rare disease is defined to affect less than 5/10,000) and 399 because they had no prevalence information. An additional thirty seven trials studied conditions with prevalence recorded as “Unknown”. These were excluded from the analysis reported in the main paper, which is therefore based on a total of 1567 trials, but are included in analyses reported in the Appendices.

### Statistical analyses

The characteristics of trials of diseases in each prevalence category were summarised, either as frequencies and percentages for categorical data or means and standard deviations for continuous data. In addition to the characteristics listed in Section Aggregate analysis of clinicaltrials.gov database (AACT) above, the duration of collection of primary outcome were calculated, from the date that enrolment to the protocol begins to either the actual completion date or anticipated date where trials were ongoing. Phase 2/3 trials were grouped with phase 3 trials; these will be collectively referred as phase 3 henceforth.

Analysis of variance and linear regression models were used to investigate the association between prevalence and trial characteristics and the sample size. This was the actual sample size for completed trials where this was available and the anticipated sample size for non-completed trials. As skewness of the distribution of the sample size was anticipated, the dependent variable in the analyses was taken to be the logarithm of the trial sample size. The fitted mean sample size and its 95% confidence interval (CI) were then back transformed by taking the exponential of the fitted values. The independent variables used for the regression analyses are marked with * in Table 2 (see Appendix 4). A few variables were not used for this analysis: whether or not the trial accepts healthy volunteers, whether or not that a trial with an FDA-regulated intervention was a FDAAA801 clinical trial, masking, allocation, primary endpoint, overall recruitment status and primary completion duration. These variables were not included in the regression models because the number of trials with healthy volunteers was very small (<2%; variable, whether or not the trial accepts healthy volunteers), because FDAAA801 trials are a subset of those for which the intervention was FDA regulated, because masking, allocation, primary endpoint were highly collinear with phase, because overall recruitment status was not a design feature and because primary completion duration was closely related to sample size. It was expected that prevalence class and phase of study would influence the choice of sample size and so to explore the effect of other covariates, these were added in turn to a model that included prevalence class, phase of study and the interaction of these two covariates. Covariates were considered to have a significant effect on the sample size if they were significant at the *p* < 0.05 level. This relatively stringent condition was used as we were more concerned with determining which factors are associated with sample size than in prediction or adjusting for all possible factors. The effects of prevalence class and phase of study were then considered based on both an unadjusted model and a model adjusting for all other significant covariates. Pairwise comparison were used to investigate further the difference between levels of a covariate.

## Results

### Trial characteristics

Table 2 (see Appendix 4) shows characteristics and features of the 1567 trials for each prevalence class. The number of trials studying conditions with prevalence <1/1,000,000, 1–9/1,000,000, 1–9/100,000, and 1–5/10,000 were 19 (1.21%), 126 (8.04%), 791 (50.48%), and 631 (40.27%), respectively. Of the 1567 trials, 1361 (87%) were conducted in one country only; US only (*m* = 823, 53%) or one European country only (*m* = 538, 34%). This seems to suggest that trials were still frequently conducted in one country despite the appeal of accessibility to a larger pool of eligible patients in multi-nation trials.

**Fig. 1:**
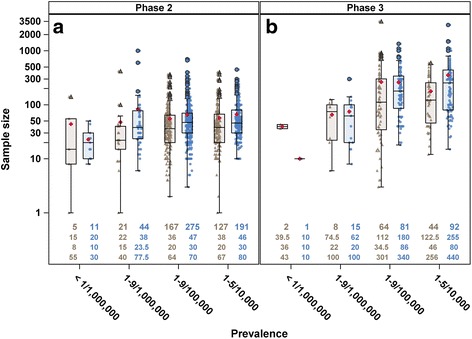
Jittered boxplot of (**a**) phase 2 and (**b**) phase 3 trials with either actual (*brown triangle*) or anticipated (*blue dot*) sample size by prevalence class. Each symbol represents one observation and the mean sample size is indicated by the red diamond. Number of trials contributing to the plot is given at the top row, median sample size in the second row, first quartile in the third row and third quartile in the last row of the bottom of each boxplot

Figure 1 shows the sample size of phase 2 trials (Fig. 1 (a)), and phase 3 (combined phase 2/3 and phase 3) trials (Fig. 1 (b)) for each prevalence class separately for completed and ongoing trials. Within each prevalence class the small plotted symbols represent the observed data, with triangles giving actual and dots giving anticipated sample sizes. The large plotted red diamonds give the mean values while the box plots show the median, first and third quartiles. The whisker shows the minimum (maximum) observation above (below) the 1.5 times the interquartile range. Note that the lower whisker appears to include a wider range because the y-axis is in log-scale.

As expected there were more phase 2 than phase 3 trials, and the median sample size for phase 3 trials was higher than those in phase 2. Figure 1 shows that the median of actual sample size from completed trials was generally lower than the median of anticipated size and that there is a wide spread of actual/anticipated sample sizes. Figure 1 (a) shows sample sizes for phase 2 trials, indicating that there was no strong association between prevalence and sample size. About 75% of the rarer diseases (<1/1,000,000 and 1–9/1,000,000) trials had size less than 50; actual median size, 15 (interquartile range, IQR, 8–55), anticipated median size, 20 (IQR, 10–30) for prevalence <1/1,000,000 and actual and anticipated median sizes for prevalence 1–9/1,000,000 were 22 (IQR, 15–40) and 38 (IQR, 23.5–77.5), respectively. This was also the case for more than half of the trials of less rare diseases: 1–9/100,000; actual and anticipated median sizes were 36 (IQR, 20–64) and 47 (IQR, 30–70), respectively; and 1–5/10,000; actual and anticipated median sizes were 38 (IQR, 20–67) and 46 (IQR, 30–80). The third quartile of the boxplots was below 100 showing that less than 25% trials across different classes of prevalence had size greater than 100.

Figure 1 (b) shows sample sizes for phase 3 trials. There were fewer number of phase 3 rarer diseases trials (*m* = 26) than phase 2 (*m* = 81). Here there is slightly more indication that the sample size is larger for trials in less rare diseases. The actual and anticipated median sizes for diseases with prevalence <1/1,000,000 were 39.5 (IQR, 36–43) and 10 (IQR, 10–10), respectively; for diseases with prevalence 1–9/1,000,000 were 74.5 (IQR, 22–100) and 62 (IQR, 20–100), respectively; for diseases with prevalence 1–9/100,000 were 112 (IQR, 34.5–301) and 180 (IQR, 86–340), respectively; and for diseases with prevalence 1–5/10,000 were 122.5 (IQR, 46–256) and 255 (IQR, 80–440), respectively.

### Main analysis

Covariates that were found to be statistically significantly related to sample size were inclusion criteria gender and age, whether or not the trial had a DMC, whether or not the intervention was FDA regulated, intervention model, trial regions, number of countries participating in the trial, year that enrolment to the protocol begins and number of treatment arms (see Table 3 in Appendix 5). Trials that recruited females only had the highest fitted mean size (58.12; 95% confidence interval, CI, 44.23–76.38) whilst those that recruited male only had the lowest (21.09; 95% CI, 14.04–31.69). This effect may be confounded by the indication or disease that affects females or males only but not both. Unsurprisingly, trials for children only (term new born infants to adolescents up to 18 years) had the smallest estimated size (36.29; 95% CI, 28.03–46.99). We might have expected the age group 18–65 years old (adults only) to have the most patients and thus trials for this age range would be the largest. However, the estimated mean was 58.49 (95% CI, 45.34–75.45) whereas trials for elderly only (65 years or older) had the largest size (89.17; 95% CI, 58.86–135.08). Trials not of an FDA regulated intervention were marginally larger, mean, 52.30 (95% CI, 43.72–62.56) compare to those with FDA regulated intervention, 46.20 (95% CI, 38.77–55.06). Trials with DMC had larger size (52.43; 95% CI, 44.06–62.38 vs. 42.95; 95% CI, 35.92–51.36). Trials with a factorial design had the largest sample size (139.83; 95% CI, 56.36–346.91) compared to parallel assigned trials (71.38; 95% CI, 60.45–84.28), single group (34.28; 95% CI, 29.05–40.46) and crossover trials (28.63; 95% CI, 21.84–37.53). There was no significant relation between lead sponsor and sample size. Trials conducted in the EU had the largest sample size (71.63; 95% CI, 55.99–91.64) followed by trials conducted in one European country only (49.77; 95% CI, 41.55–59.62), in the US and EU (46.29; 95% CI, 35.40–60.53) and in the US only had the smallest (42.50; 95% CI, 35.57–50.79). There seemed to be a slight decrease of sample size for trials in which enrolment started from year 2005 than those before then. Number of treatment arms in a trial affects the sample size required with trials with more arms tending to have larger sample size. The estimated increase in sample size per arm was 14%.

**Fig. 2:**
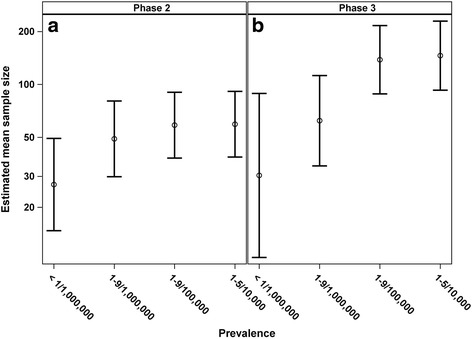
Fitted mean of sample size and 95% confidence interval back transformed from logarithmic values by class of prevalence and phase of trial adjusted for interaction between prevalence, phase of study and the interaction between prevalence and phase, adjusted for gender, age, whether or not the trial had a DMC, whether or not the intervention was FDA regulated, intervention model, trial regions, number of countries participating in the trial, year that enrolment to the protocol begins and number of arms

**Fig. 3:**
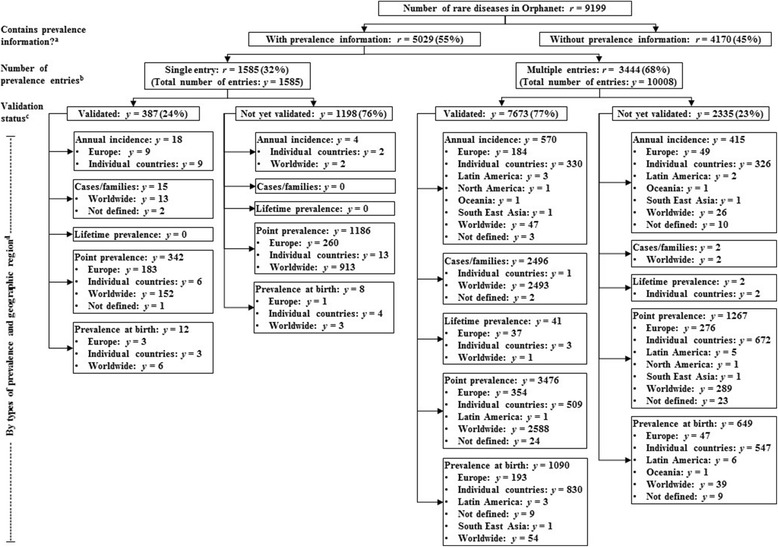
A breakdown of epidemiological data of rare diseases in Orphadata. *a* Whether or not the rare disease has any prevalence data; *b* Number of prevalence data entries per rare disease (extracted from literature); *c* Whether or not the given prevalence value is validated; *d* The subtype of prevalence information by geographical area

**Fig. 4:**
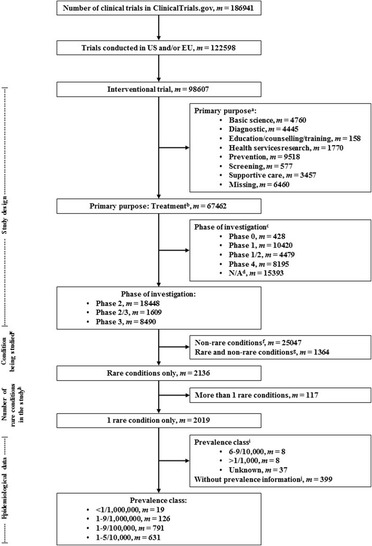
A breakdown of trials in ClinicalTrials.gov included in final analyses. *a* The primary purpose of the trial; *b* A treatment-purpose trial is to evaluate one or more interventions for treating a disease, syndrome or condition; *c* Phase of investigation as defined by the US FDA; d For trials that do not involve drug or biologic products (e.g., behavioural interventions); *e* Primary disease or condition being studied in the trial. The terms of the conditions should follow the National Library of Medicine’s Medical Subject Headings (MeSH) where possible; *f* Trials studying non-rare disease(s)/condition(s); *g* Trials studying both rare and non-rare disease(s)/condition(s); *h* Number of rare disease(s)/condition(s) being studied in a trial. *i* Estimated prevalence from Orphanet; *j* The rare disease/condition has no prevalence data from Orphanet

Figure 2 shows the fitted mean sample size (back transformed from logarithmic values), together with 95% confidence intervals, for trials in different prevalence class and phase of trial after adjusting for the covariates listed in the preceding paragraph. Effects of prevalence and phase were statistically significant after adjusting for the other covariates, *p* values were <0.0001 and 0.0006, respectively, and the interaction between prevalence and phase was close to significance, *p* = 0.0828. It is interesting to note from Fig. 2 that there is no apparent effect of prevalence in phase 2 trials. From Table 1, the fitted mean sample size for diseases with prevalence <1/1,000,000 in phase 2 was the lowest, 26.96 (95% CI, 14.74–49.31). Fitted mean sizes across the other prevalence classes were similar; 49.04 (95% CI, 29.87–80.51), 58.70 (95% CI, 38.14–90.32) and 59.42 (95% CI, 38.62–91.43) for prevalence 1-9/1,000,000, 1-9/100,000 and 1–5/10,000, respectively. There is an apparent effect of prevalence in phase 3 trials (Fig. 2), where the trial size for diseases that are in the slightly less rare (1–9/100,000 and 1–5/10,000 prevalence classes) tended to be larger than those for the rarer diseases (<1/1,000,000 and 1–9/1,000,000 prevalence classes). The fitted mean sample sizes were 30.37 (95% CI, 10.37–88.92), 62.21 (95% CI, 34.40–112.49), 138.28 (95% CI, 88.39–216.34) and 145.86 (95% CI, 92.79–229.30) for prevalence <1/1,000,000, 1–9/1,000,000, 1–9/100,000 and 1–5/10,000, respectively. In pairwise comparisons between <1/1,000,000, and 1–9/100,000 and 1–5/10,000, the differences were statistically significant, *p* = 0.0047 and 0.0039, respectively (results not shown). The differences between 1–9/1,000,000, and 1–9/100,000 and 1–5/10,000 were also statistically significant, *p* < 0.0001 in both pairwise comparisons (result not shown). Note that the sizes for rarer diseases (<1/1,000,000 and 1–9/1,000,000 prevalence classes) in phase 3 were also similar to those in phase 2. Although the wide variation in sample sizes and the relatively small numbers of trials for some prevalence classes leads to wide confidence intervals, similar conclusions can be drawn to those given above based on Fig. 1 and the fitted regression model with class of prevalence, phase of trial and the interaction between prevalence and phase (Table 1).

**Table 1:** Fitted mean of sample size and 95% confidence interval back transformed from logarithmic values, type III F statistic and the corresponding *p* value of the effect of class of prevalence on sample size adjusting for phase and interaction between prevalence and phase, and without adjustment for other covariates and with adjustment for other covariates^a^

Characteristics		Without adjustment for other covariates					With adjustment for other covariates				
		No. of trials, *m*	Fitted mean	95% CI	*F* test	*p* value	No. of trials, *m*	Fitted mean	95% CI	*F* test	*p* value
Prevalence class					17.38	<.0001				20.73	<.0001
<1/1,000,000		19	21.21	(11.63–38.67)			18	28.61	(14.43–56.73)		
1–9/1,000,000		88	40.57	(32.18–51.14)			66	55.23	(33.76–90.37)		
1–9/100,000		587	74.19	(67.71–81.29)			483	90.09	(58.66–138.36)		
1–5/10,000		454	86.37	(78.32–95.25)			359	93.10	(60.58–143.08)		
Phase of investigation ^b^					21.56	<.0001				324.97	<.0001
Phase 2		841	32.84	(28.61–37.69)			677	46.34	(29.86–71.90)		
Phase 3		307	71.50	(53.05–96.38)			249	78.57	(47.48–130.02)		
Prevalence × Phase					6.40	0.0003				2.75	0.0415
Phase 2	<1/1,000,000	16	18.05	(11.20–29.09)			15	26.96	(14.74–49.31)		
	1–9/1,000,000	65	35.23	(27.80–44.65)			48	49.04	(29.87–80.51)		
	1–9/100,000	442	41.52	(37.92–45.47)			365	58.70	(38.14–90.32)		
	1–5/10,000	318	44.04	(39.57–49.02)			249	59.42	(38.62–91.43)		
Phase 3	<1/1,000,000	3	24.92	(8.28–75.06)			3	30.37	(10.37–88.92)		
	1–9/1,000,000	23	46.71	(31.37–69.56)			18	62.21	(34.40–112.49)		
	1–9/100,000	145	132.56	(113.12–155.35)			118	138.28	(88.39–216.34)		
	1–5/10,000	136	169.39	(143.80–199.53)			110	145.86	(92.79–229.30)		

^a^Gender, age, whether or not the trial had a DMC, whether or not the intervention was FDA regulated, intervention model, trial regions, number of countries participating in the trial, year that enrolment to the protocol begins and number of arms

^b^As defined by the US FDA for trials involving investigational new drugs

The R-squared statistic, an indication of the proportion of variability of fitted log sample size by the prevalence, phase, interaction between prevalence and phase and the other covariates was 0.4184. This is small despite the large number of regressors in the model, suggesting that there appears to be a lot of unexplained variability.

### Sensitivity analysis

About one third of the trials (*m* = 587, 37%) used parallel assignment and about half (*m* = 792, 51%) used single group assignment. We performed sensitivity analyses with parallel 2-arm trials only and single group assignment (1-arm) trials only to investigate the effect of prevalence and phase of study adjusted by covariates on sample size.

For the analysis of parallel 2-arm trials only, we also included the types of arm (experimental, active comparator, placebo comparator, sham comparator, no intervention or others) as one of the covariates that may be associated with sample size. The possible combinations of 2-arm trials are: experimental vs. placebo (*m* = 88), experimental vs. standard (active comparator, no intervention, others) (*m* = 139), experimental vs. experimental (*m* = 49) and non-experimental vs. non-experimental (*m* = 75). There were 354 parallel 2-arms trial and Table 4 (Appendix 6) shows covariates that were statistically significant related to sample size: gender, age, whether or not the trial had a DMC, trial regions, number of countries participating in the trial and types of arm. The fitted sample size for trials where the experimental arm vs. the standard arm was the highest, mean 106.78 (95% CI, 83.63–136.33). Fitted mean sample sizes for trials across the other types of 2-arm were very similar; 52.82 (95% CI, 41.54–67.17), 53.44 (95% CI, 39.86–71.63) and 59.78 (95% CI, 45.57–78.43) for experimental vs. placebo, experimental vs. experimental and non-experimental vs. non-experimental, respectively. Effects of prevalence class and phase of trial after adjusting for all the significant covariates on parallel 2-arm trials were statistically significant, *p* = 0.0004 and 0.0036, respectively (see, Table 5, Appendix 7). However, the interaction between prevalence and phase was not, *p* = 0.3727.

**Fig. 5:**
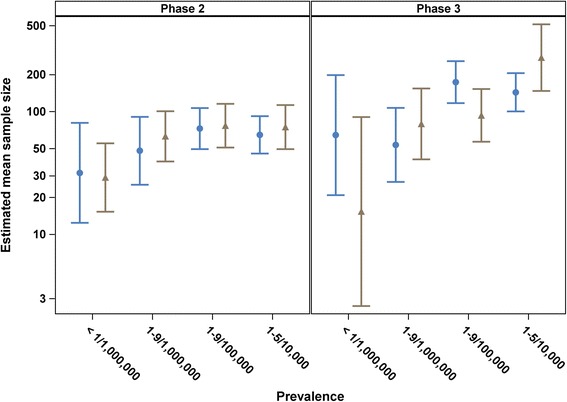
Fitted mean of sample size and 95% confidence interval back transformed from logarithmic values by class of prevalence and phase of trial adjusted for interaction between prevalence, phase of study and the interaction between prevalence and phase for parallel 2-arm trials only (*blue*) and single group (1-arm) trials only (*brown*). Both fitted models were adjusted for other covariates (see Table 5)

There were 527 single group (1-arm) trials and Table 6 (Appendix 8) shows that lead sponsor, trial regions, number of countries participating in the trial and year that enrolment to the protocol began were significantly related to sample size. Only effects of prevalence and interaction between prevalence and phase were significant after adjusting for all significant covariates (*p* < 0.0001 and 0.0013, respectively, see Table 5). Effects of phase of study was not statistically significant (*p* = 0.2873). Overall, we observed similar trend where sample size is affected by prevalence where as the prevalence increases, mean sample size increases with a more noticeable difference in phase 3 trials (see Fig. 5 in Appendix 9).

## Discussion

We found that a majority of trials were conducted in one country only regardless of the disease prevalence. This is slightly surprising given the opportunity in multi-nation trials to recruit more patients. Further investigation may be necessary to understand why multi-nation trials were not conducted more frequently.

We also found that the actual sample size for completed trials was generally smaller than the anticipated trial size for ongoing trials. This supports results shown by Bell and Tudur Smith where there were more rare disease trials (35%) with actual enrolment of 50 or less and 29% of rare disease trials with anticipated enrolment of 50 or less [5]. This could be indicative of an ambition to complete large trials in rare disease populations that are difficult to achieve in practice.

Sample sizes for trials in rare diseases were statistically significantly related to gender, age, whether or not the trial had a DMC, whether or not the intervention was FDA regulated, intervention model, trial regions with at least one participating centre, number of countries participating in the trial, year that enrolment to the protocol began and number of treatment arms.

Trials enrolling males only were on average smaller than those that enrolled either females only or both sexes. Trials enrolling females only had slightly larger size than those that enrolled both sexes but this was not statistically significantly different. We expected that trials enrolling males only and females only to have smaller size because when the eligibility criteria is restrictive, the population is more homogeneous and less variable in effectiveness, thus smaller sample size may be sufficient. Further inspections revealed that of the 79 trials with females only, 78% (*m* = 62) of them were in phase 2 and 89% (*m* = 70) were for diseases with prevalence 1-5/10,000. There were only 25 trials with males only and 76% (*m* = 19) were in phase 2 and only 36% (*m* = 9) were for diseases with prevalence 1-5/10,000. The small number of less rare diseases for males might have influenced the average sample size in male-only trials as shown in Table 7 (Appendix 10), a list of diseases by phase for females and males only. Of note is that most of these trials were in diseases that affect one sex only; all of the male-only trials were X-linked disorders whereas almost all of the female-only trials affected females only. A few of these trials were in disorders for pregnant women only. Further research is necessary to investigate and identify other factors that could explain this difference.

Similarly, we expected trials enrolling various age groups to have larger sample sizes than those that recruited children only, adults only or elderly only because by expanding the sampling pool more patients could be recruited. However, on average trials recruiting multiple age groups were slightly smaller than adults-only and elderly-only trials.

Unsurprisingly, trials with factorial design had larger sample size than single group and crossover trials since in a factorial design a few combinations of interventions are tested at the same time. Diseases that employed the factorial design had prevalence greater than 1/100,000 (the less rare diseases) suggesting that sophisticated designs could be used when possible. However, the most frequently used intervention model for the rarer diseases (prevalence <1/100,000) was single group assignment and the average sample size was less than 35. The levels of evidence from these trials may not be as high quality as the gold standard RCT. The EMA has indicated that prevalence of disease could constrain the design, conduct and analysis of trials for small populations and the EMA/CHMP guideline suggested that novel approaches could be considered in situations when it is difficult to recruit large number of patients [3]. This in turn presents a challenge of developing new methodology for trials in small populations. In response to this challenge, three collaborative research projects (Asterix, IDeAl and InSPiRe) are working on methods for clinical trials in the small population setting [13].

The main analysis and sensitive analyses with parallel 2-arm trials only and single group (1-arm) trials only showed that generally, the mean sample size was affected by prevalence where mean sample size increases as prevalence increases. The increase was noticeably larger in phase 3 trials compare to phase 2. However, due to small number of trials in some classes, it is difficult to make comparisons.

The generalisability of the results obtained in this study rely on the extent to which trials included in the database are representative. Although institutions such as the International Committee of Medical Journal Editors (ICMJE) require certain studies to be registered either in ClinicalTrials.gov or other equivalent registries [14, 15], it seems likely that certain types of trials are more likely to be registered, especially, efficacy trials in serious or life-threatening diseases with investigational new drugs regulated by the FDA and EMA. This is a strength of this research as we concentrated on interventional phase 2 and/or 3 trials where there would be better coverage. However, phase 2 and/or 3 trials taking place in EU site(s) initiated after 2011 may not be registered in ClinicalTrials.gov but in the EU Clinical Trials Register which was launched on 22 March 2011 [16].

A limitation with our study is the potential selection bias because we included only trials conducted in the US and/or the EU. This is a necessary measure to exclude trials studying diseases with low prevalence in the US/EU but high prevalence elsewhere. For example, there was a multi-centre interventional trial on tuberculosis with locations in the US, United Kingdom and Peru. The annual incidence in these countries are 1-9/100,000, 1-5/10,000 and >1/1,000, respectively [8, 17].

Another possible limitation with our study is that we considered a condition to be rare if information on prevalence was listed in Orphadata. This database is updated on a regular basis and some conditions may have been missed out or with no prevalence information. Table 8 in Appendix 11 provides a list of trials in the AACT database where the conditions studied were listed in Orphadata but for which no value of prevalence is given. Prevalence of some diseases changes over time and because the prevalence information in Orphadata is updated regularly, old prevalence data are not retained. This presents a weakness to the study as trials studying rare diseases prior to 2016 were assumed to have updated prevalence.

As explained in the methods section, we have used point prevalence to classify diseases into prevalence classes where this is available. In some cases, some other measure of prevalence has been used. In this project diseases are classified into groups according to their prevalence value and because of categorising continuous variable we have lost some information. However, this is a necessary pragmatic approach so that ultra rare diseases where only number of cases/families were known could be included in the analysis. In these diseases it is unknown which denominator should be used to calculate the prevalence value but they could be classified as having prevalence <1/1,000,000, as is the practice in Orphadata. Our results depend to some extent on the choice of types of prevalence used but as the results presented are based on means from a number of studies, it is likely that conclusions are relatively robust.

In our analysis we have grouped trials described as phase 2/3 by investigators with trials described as phase 3. This is a reasonable assumption because the eventual objective of both phase 2/3 and phase 3 trials is to test the study hypothesis whether or not the treatment is more effective with a plan to subsequently submit for regulatory approval. However, there may have been inconsistency in data entry by investigators with the definition given by US FDA. This is likely to introduce systematic bias. Theses inconsistencies are difficult to rectify as the registry does not require investigators to give details on the design and sample size calculation where detailed examinations could be performed to check if the objective of the design correspond to the US FDA definition.

The number of patients eligible for trials may also depend on whether the rare condition is acute or life threatening, so that only new cases can be recruited, or chronic, when it may be possible to sample from a larger population depending on the prevalence rather than the incidence rate. Further work should investigate the association of acuteness/chronicity of the condition on the trial sample size.

We have focussed attention on the sample size of trials in rare diseases. The AACT database also contains additional data, for example on trial design features such as the intervention model (crossover, factorial, parallel or single group assignment), masking (double blind, single blind or open label), allocation (randomized, non-randomized), primary endpoint (e.g., efficacy, safety, pharmacodynamics, pharmacokinetics) and number of interventions in a trial. These might also vary with disease prevalence among rare disease trials. Investigation of such effects could be the subject of further work.

## Conclusions

This study has investigated sample sizes for clinical trials in rare diseases using data from the AACT database from ClinicalTrials.gov and prevalence data from the Orphadata databases from Orphanet. These databases provide rich resources to understand and characterise clinical trials studying rare diseases or conditions. The inventory of rare disease in Orphanet is updated on a regular basis and the prevalence and other information of the diseases are based on published scientific articles.

We have limited our analyses to phase 2, phase 2/3 or phase 3 trials with treatment as the primary purpose conducted in the US and/or EU (member states of the EU and associated countries). We found that where were very few multi-nation trials suggesting that the opportunities to conduct larger or ‘adequately’ size trials were underused. We also found that the fitted mean sample sizes for rare disease trials do differ slightly between prevalence classes (the interaction between prevalence and phase was close to significance) with slightly larger trials conducted in diseases with higher prevalence. This effect was most noticeable in phase 3 trials where sample sizes for the rarer diseases are similar to those for phase 2 trials, but are larger than those for phase 2 trials in the less rare of the rare diseases considered.

## Appendix 2

### Merging of AACT and Orphadata

Data from AACT is supplied as delimited text files and Orphadata were downloaded as Extensible Markup Language (XML) files. A database schema was first derived from evaluating the content of each text files in order to identify the database requirements. A SQL Server Integration Services (SSIS) program was then created to map the text files to the database schema and to extract and load the data into the database. A random sample of data from each text file was used to check against the database to verify data integrity. The Orphadata database schema was modified to include two additional tables to store disorder and prevalence data. An Extensible Stylesheet Language Transformation (XSLT) was created to parse the XML to create SQL insert statements for each disorder and prevalence entry. Data integrity was checked again by taking a random sample from the original XML files to compare against the data inserted into the database.

For each trial, the prevalence of the disease in the countries where the trial took place was identified. If there was more than one prevalence entries for the disease in the trial location, the prevalence was used based on the following variables (in decreasing order of preference):

1. Validation status: (i) validated, and (ii) not yet validated.
2. Type of prevalence: (i) point prevalence, (ii) lifetime prevalence, (iii) prevalence at birth, (iv) annual incidence, and (v) cases/families.

If no prevalence information was available for the disease in the trial location then the prevalence for a neighbouring country or another country from the same geographic region was used. For trial conducted in US only, prevalence from that country was used. If there was no data from the US, data from the following was used (in decreasing order): North America, Europe (region), individual European country, Worldwide and other countries. For trials conducted in a single European country, data from the following locations were used (in decreasing order): the European country where the trial was conducted, Europe (as a region), other European countries, US, North America, Worldwide and other countries. For trials conducted in Europe, prevalence from Europe was used. If that was not available data from other locations were used, in decreasing order: other European countries or US, North America, Worldwide and other countries. Finally, for trials conducted in both US and Europe, prevalence data from both US and Europe were used and then ordered by validation status and type of prevalence. If either of these were not available data from other geographical area, in decreasing order, were used: European countries, North America, Worldwide and other countries. In all cases when there were more than one prevalence information, they were ordered by validation status and type of prevalence. Note that if only the number of cases/families was recorded, the prevalence was assumed to be <1/1,000,000.

## Appendix 4

**Table 2:** Characteristics of rare disease trials conducted in the United States (US) and/or European Union (EU) by class of prevalence^a^. Data are number of trials, *m* (%), or mean (standard deviation, SD)

	Class of prevalence									
	<1/1,000,000 (*m* = 19)		1–9/1,000,000 (*m* = 126)		1–9/100,000 (*m* = 791)		1–5/10,000 (*m* = 631)		Unknown (*m* = 37)	
	*m*	(%)	*m*	(%)	*m*	(%)	*m*	(%)	*m*	(%)
Inclusion criteria										
*Gender										
Both	17	(89)	119	(94)	772	(97)	505	(80)	33	(89)
Female	.	.	5	(3)	6	(<1)	111	(17)	1	(2)
Male	2	(10)	2	(1)	13	(1)	15	(2)	3	(8)
Accepts healthy volunteers?										
Yes	1	(5)	2	(1)	11	(1)	7	(1)	.	.
No	18	(94)	124	(98)	778	(98)	620	(98)	37	(100)
Missing	.	.	.	.	2	(<1)	4	(<1)	.	.
*Age categories ^b^										
Children only	2	(10)	11	(8)	65	(8)	42	(6)	2	(5)
Adults only	1	(5)	3	(2)	45	(5)	68	(10)	1	(2)
Elderly only	.	.	.	.	24	(3)	9	(1)	.	.
Children to elderly	9	(47)	36	(28)	107	(13)	69	(10)	11	(29)
Children and adults	2	(10)	16	(12)	42	(5)	31	(4)	.	.
Adults to elderly	5	(26)	60	(47)	508	(64)	412	(65)	23	(62)
Study designs										
Phase of investigation ^c^										
Phase 2	16	(84)	92	(73)	602	(76)	450	(71)	28	(75)
Phase 2/3	3	(15)	6	(4)	30	(3)	23	(3)	4	(10)
Phase 3	.	.	28	(22)	159	(20)	158	(25)	5	(13)
*Has DMC? ^d^										
Yes	7	(36)	51	(40)	401	(50)	258	(40)	17	(45)
No	11	(57)	37	(29)	224	(28)	207	(32)	11	(29)
Missing	1	(5)	38	(30)	166	(20)	166	(26)	9	(24)
*FDA regulated intervention? ^e^										
Yes	10	(52)	67	(53)	438	(55)	246	(38)	19	(51)
No	9	(47)	39	(30)	271	(34)	275	(43)	13	(35)
Missing	.	.	20	(15)	82	(10)	110	(17)	5	(13)
Is Section 801? ^f^										
Yes	10	(52)	59	(46)	389	(49)	213	(33)	14	(37)
No	.	.	8	(6)	45	(5)	28	(4)	3	(8)
Missing	9	(47)	59	(46)	357	(45)	390	(61)	20	(54)
*Intervention model										
Crossover assignment	.	.	7	(5)	43	(5)	27	(4)	1	(2)
Factorial assignment	.	.	.	.	5	(<1)	4	(<1)	.	.
Parallel assignment	7	(36)	32	(25)	284	(35)	264	(41)	7	(18)
Single group assignment	12	(63)	79	(62)	425	(53)	276	(43)	28	(75)
Missing	.	.	8	(6)	34	(4)	60	(9)	1	(2)
Masking										
Double blind	5	(26)	19	(15)	153	(19)	139	(22)	7	(18)
Single blind	1	(5)	1	(<1)	12	(1)	11	(1)	1	(2)
Open label	13	(68)	99	(78)	601	(75)	430	(68)	28	(75)
Missing	.	.	7	(5)	25	(3)	51	(8)	1	(2)
Allocation										
Randomized	7	(36)	35	(27)	320	(40)	319	(50)	9	(24)
Non-randomized	.	.	37	(29)	183	(23)	108	(17)	11	(29)
Missing	12	(63)	54	(42)	288	(36)	204	(32)	17	(45)
Endpoint classification										
Efficacy study	3	(15)	30	(23)	199	(25)	151	(23)	9	(24)
Safety/efficacy study	14	(73)	77	(61)	467	(59)	366	(58)	27	(72)
Safety study	1	(5)	4	(3)	39	(4)	16	(2)	.	.
Bio-equivalence study	.	.	.	.	3	(<1)	1	(<1)	.	.
Pharmacodynamics study	.	.	.	.	.	.	3	(<1)	.	.
Pharmacokinetics study	.	.	1	(<1)	3	(<1)	1	(<1)	.	.
Pharmacokinetics/dynamics study	.	.	1	(<1)	.	.	2	(<1)	.	.
Missing	1	(5)	13	(10)	80	(10)	91	(14)	1	(2)
Interventions, facilities and authorities										
*Lead sponsor										
US Federal	.	.	2	(1)	.	.	6	(<1)	.	.
Industry	10	(52)	35	(27)	208	(26)	185	(29)	9	(24)
NIH	.	.	12	(9)	47	(5)	21	(3)	3	(8)
Other	9	(47)	77	(61)	536	(67)	419	(66)	25	(67)
*Trial location										
US only	6	(31)	77	(61)	485	(61)	255	(40)	17	(45)
EU only	3	(15)	7	(5)	28	(3)	86	(13)	2	(5)
US and EU	4	(21)	6	(4)	38	(4)	34	(5)	2	(5)
Single European country	6	(31)	36	(28)	240	(30)	256	(40)	16	(43)
*No. of countries where trials run										
*m*	19		126		791		631		37	
Mean	1.53		1.43		1.25		1.70		1.35	
Standard deviation, SD	0.77		1.52		1.10		2.06		1.27	
*No. of arms										
*m*	19		106		700		523		29	
Mean	1.53		1.42		1.60		1.66		1.38	
SD	0.84		0.69		0.85		0.72		0.68	
*Year that enrolment to the protocol begins										
<1990	.	.	1	(<1)	2	(<1)	1	(<1)	1	(2)
1990–1999	.	.	6	(4)	40	(5)	47	(7)	2	(5)
2000–2004	1	(5)	27	(21)	117	(14)	90	(14)	4	(10)
2005–2009	5	(26)	42	(33)	266	(33)	263	(41)	15	(40)
2010–2014	11	(57)	49	(38)	328	(41)	210	(33)	14	(37)
2015 and after	2	(10)	.	.	25	(3)	14	(2)	1	(2)
Missing	.	.	1	(<1)	13	(1)	6	(<1)	.	.
Overall recruitment status										
Active, not recruiting	2	(10)	17	(13)	131	(16)	103	(16)	3	(8)
Completed	7	(36)	44	(34)	308	(38)	258	(40)	14	(37)
Enrolling by invitation	.	.	3	(2)	8	(1)	3	(<1)	.	.
Not yet recruiting	2	(10)	6	(4)	26	(3)	22	(3)	3	(8)
Recruiting	8	(42)	35	(27)	209	(26)	152	(24)	12	(32)
Suspended	.	.	2	(1)	1	(<1)	7	(1)	.	.
Terminated	.	.	13	(10)	84	(10)	66	(10)	4	(10)
Withdrawn	.	.	6	(4)	24	(3)	20	(3)	1	(2)
Primary completion duration (years) ^g^										
*m*	19		110		713		534		30	
Mean	2.95		4.16		3.81		3.46		3.27	
SD	2.7		3.35		2.88		2.33		1.75	

* Covariates considered in the ANOVA/linear regression model as described in Section Statistical analyses

^a^ Member states of the EU and associated countries

^b^ Minimum and maximum age groups are mutually exclusive. Children, term new born infants to adolescents up to 18 years; adults, 18–64 years old; and elderly, 65 years or older

^c^ As defined by the US FDA for trials involving investigational new drugs

^d^ Indicate whether or not a data monitoring committee (DMC) has been appointed for this study

^e^ Indicate whether or not the trial includes an intervention subject to US Food and Drug Administration regulation under section 351 of the Public Health Service Act or any of the following sections of the Federal Food, Drug and Cosmetic Act: 505, 510(k), 515, 520(m), and 522

^f^ Whether the FDA regulated intervention is an “applicable clinical trial” as defined in US Public Law 110–85, Title VIII, Section 801

^g^ The difference between enrolment date and the actual primary completion date where the final subject was examined or received the intervention for the purpose of data collection for the primary outcome or anticipated date where trials were ongoing

## Appendix 5

**Table 3:** Fitted mean of sample size and 95% confidence interval (CI) back transformed from logarithmic values, type III F statistic and the corresponding *p* value of the effect of the covariate on sample size adjusted by class of prevalence, phase of trial and interaction between prevalence and phase

Characteristics	No. of trials, *m*	Fitted mean	95% CI	*F* test	*p* value
Inclusion criteria					
Gender	1148			10.61	<.0001
Both		49.30	(41.85–58.08)		
Female		58.12	(44.23–76.38)		
Male		21.09	(14.04–31.69)		
Age categories ^a^	1148			4.52	0.0004
Children only		36.29	(28.03–46.99)		
Adults only		58.49	(45.34–75.45)		
Elderly only		89.17	(58.86–135.08)		
Children and adults		51.94	(39.74–67.87)		
Children to elderly		44.98	(36.88–54.85)		
Adults to elderly		50.93	(42.66–60.80)		
Study designs					
Has DMC? ^b^	963			9.94	0.0017
No		42.95	(35.92–51.36)		
Yes		52.43	(44.06–62.38)		
FDA regulated intervention? ^c^	1076			4.09	0.0433
No		52.30	(43.72–62.56)		
Yes		46.20	(38.77–55.06)		
Intervention model	1114			56.55	<.0001
Crossover assignment		28.63	(21.84–37.53)		
Factorial assignment		139.83	(56.36–346.91)		
Parallel assignment		71.38	(60.45–84.28)		
Single group assignment		34.28	(29.05–40.46)		
Interventions, facilities and authorities					
Lead sponsor	1148			1.53	0.2041
US Federal		68.43	(30.81–151.99)		
Industry		46.20	(38.50–55.43)		
NIH		65.80	(44.80–96.65)		
Other		49.20	(41.46–58.38)		
Trial location	1148			7.68	<.0001
US only		42.50	(35.57–50.79)		
EU only		71.63	(55.99–91.64)		
US and EU		46.29	(35.40–60.53)		
Single European country		49.77	(41.55–59.62)		
No. of countries involved in the trial	1148	1.07 ^d^	(1.04–1.11)	16.07	<.0001
Year that enrolment to the protocol begins	1141			3.14	0.0081
<1990		131.48	(34.06–507.57)		
1990–1999		57.83	(41.44–80.69)		
2000–2004		63.10	(50.12–79.44)		
2005–2009		44.61	(37.31–53.34)		
2010–2014		48.72	(40.92–58.01)		
2015 and after		49.89	(35.70–69.71)		
No. of arms	1085	1.39 ^d^	(1.30–1.49)	83.75	<.0001

^a^ Minimum and maximum age groups are mutually exclusive. Children, term new born infants to adolescents up to 18 years; adults, 18–64 years old; and elderly, 65 years or older

^b^ Indicate whether or not a data monitoring committee (DMC) has been appointed for this study

^c^ Indicate whether or not the trial includes an intervention subject to US Food and Drug Administration regulation under section 351 of the Public Health Service Act or any of the following sections of the Federal Food, Drug and Cosmetic Act: 505, 510(k), 515, 520(m), and 522

^d^ Estimated coefficient, the estimated increase of sample size for every unit increase of the covariate

## Appendix 6

**Table 4:** Fitted mean of sample size and 95% confidence interval (CI), type III F statistic and the corresponding *p* value of the effect of the covariate on sample size adjusted by class of prevalence, phase of trial and interaction between class and phase for parallel 2-arm trials only

Characteristics	No. of trials, *m*	Fitted mean	95% CI	*F* test	*p* value
Inclusion criteria					
Gender	354			4.66	0.0101
Both		64.21	(51.30–80.36)		
Female		105.11	(70.26–157.26)		
Male		38.08	(15.93–91.00)		
Age categories ^a^	354			2.32	0.043
Children only		44.44	(29.90–66.05)		
Adults only		75.84	(53.16–108.21)		
Elderly only		100.50	(56.18–179.79)		
Children and adults		73.17	(48.83–109.64)		
Children to elderly		56.93	(42.19–76.84)		
Adults to elderly		70.83	(55.34–90.66)		
Study designs					
Has DMC? ^b^	321			8.39	0.004
No		53.90	(41.26–70.41)		
Yes		72.29	(57.83–90.38)		
FDA regulated intervention? ^c^	347			2.31	0.1293
No		71.04	(55.51–90.92)		
Yes		61.37	(48.04–78.39)		
Interventions, facilities and authorities					
Lead sponsor	354			1.18	0.3179
US Federal		15.15	(2.72–84.37)		
Industry		62.54	(48.26–81.04)		
NIH		69.66	(28.97–167.50		
Other		67.80	(53.39–86.09)		
Trial location	354			4.53	0.0039
US only		55.61	(43.34–71.36)		
EU only		100.14	(69.12–145.08)		
US and EU		76.58	(51.07–114.83)		
Single European country		69.98	(54.48–89.90)		
No. of countries involved in the trial	354	1.07 ^d^	(1.01–1.13)	5.86	0.016
Year that enrolment to the protocol begins	354			0.83	0.5084
1990–1999		62.53	(28.01–139.61)		
2000–2004		89.96	(59.68–135.61)		
2005–2009		64.28	(50.05–82.56)		
2010–2014		65.55	(51.14–84.04)		
2015 and after		64.43	(39.24–105.79)		
Types of arms	351			18.27	<.0001
Experimental vs. Placebo		52.82	(41.54–67.17)		
Experimental vs. Standard		106.78	(83.63–136.33)		
Experimental vs. Experimental		53.44	(39.86–71.63)		
Non-experimental vs. Non-experimental		59.78	(45.57–78.43)		

^a^ Minimum and maximum age groups are mutually exclusive. Children, term new born infants to adolescents up to 18 years; adults, 18–64 years old; and elderly, 65 years or older

^b^ Indicate whether or not a data monitoring committee (DMC) has been appointed for this study

^c^ Indicate whether or not the trial includes an intervention subject to US Food and Drug Administration regulation under section 351 of the Public Health Service Act or any of the following sections of the Federal Food, Drug and Cosmetic Act: 505, 510(k), 515, 520(m), and 522

^d^ Estimated coefficient, the estimated increase of sample size for every unit increase of the covariate

## Appendix 7

**Table 5:** Fitted mean of sample size and 95% confidence interval (CI), type III F statistic and the corresponding *p* value of the effect of class of prevalence on sample size adjusting for phase and interaction between prevalence and phase with adjustment for other covariates

Characteristics		Parallel 2-arm adjusting for other covariates^a^					Single-group (1-arm) adjusting for other covariates^b^				
		No. of trials, *m*	Fitted mean	95% CI	*F* test	*p* value	No. of trials, *m*	Fitted mean	95% CI	*F* test	*p* value
Prevalence class					6.32	0.0004				8.86	<.0001
<1/1,000,000		5	45.19	(20.92–97.64)			12	21.15	(7.95–56.26)		
1–9/1,000,000		14	50.72	(29.96–85.87)			50	70.68	(43.15–115.77)		
1–9/100,000		154	112.53	(77.92–162.52)			276	84.64	(55.38–129.36)		
1–5/10,000		146	96.42	(69.18–134.38)			171	143.51	(90.06–228.69)		
Phase of investigation ^c^					8.59	0.0036				1.13	0.2873
Phase 2		167	51.75	(33.71–79.44)			455	56.97	(37.66–86.17)		
Phase 3		152	96.36	(61.60–150.74)			54	74.80	(40.53–138.05)		
Prevalence x Phase					1.05	0.3727				5.32	0.0013
Phase 2	<1/1,000,000	3	31.71	(12.42–80.93)			11	29.10	(15.32–55.27)		
	1–9/1,000,000	8	48.03	(25.40–90.80)			39	62.95	(39.30–100.84)		
	1–9/100,000	85	72.78	(49.53–106.94)			244	76.86	(50.96–115.92)		
	1–5/10,000	71	64.72	(45.54–91.98)			161	74.79	(49.48–113.06)		
Phase 3	<1/1,000,000	2	64.41	(20.93–198.25)			1	15.37	(2.61–90.47)		
	1–9/1,000,000	6	53.57	(26.74–107.31)			11	79.36	(40.81–154.35)		
	1–9/100,000	69	174.00	(117.36–257.98)			32	93.21	(56.91–152.67)		
	1–5/10,000	75	143.65	(100.29–205.75)			10	275.36	(147.46–514.19)		

^a^ Gender, age, whether or not the trial had a DMC, trial regions, number of countries participating in the trial and types of arm

^b^ Lead sponsor, trial regions, number of countries participating in the trial and year that enrolment to the protocol begins

^c^ As defined by the US FDA for trials involving investigational new drugs

## Appendix 8

**Table 6:** Fitted mean of sample size and 95% confidence interval (CI), type III F statistic and the corresponding *p* value of the effect of the covariate on sample size adjusted by class of prevalence, phase of trial and interaction between class and phase for single-assignment 1-arm trials only

Characteristics	No. of trials, *m*	Fitted mean	95% CI	*F* test	*p* value
Inclusion criteria					
Gender	527			2.58	0.0769
Both		33.38	(25.76–43.24)		
Female		24.84	(15.86–38.90)		
Male		20.84	(11.72–37.07)		
Age categories^a^	527			1.18	0.3184
Children only		27.82	(18.67–41.46)	41.46	
Adults only		35.18	(23.20–53.36)	53.36	
Elderly only		56.05	(31.69–99.15)	99.15	
Children and adults		30.30	(19.48–47.12)	47.12	
Children to elderly		33.60	(24.87–45.40)	45.4	
Adults to elderly		32.37	(24.50–42.78)	42.78	
Study designs					
Has DMC?^b^	450			0.01	0.9328
No		31.87	(24.29–41.82)		
Yes		32.11	(24.24–42.54)		
FDA regulated intervention?^c^	514			1.94	0.1647
No		35.79	(27.16–47.16)		
Yes		31.87	(24.33–41.75)		
Interventions, facilities and authorities					
Lead sponsor	527			4.49	0.004
US Federal		125.86	(49.53–319.80)		
Industry		31.99	(24.19–42.31)		
NIH		55.42	(31.66–97.02)		
Other		31.85	(24.44–41.50)		
Trial location	527			5.14	0.0017
US only		27.35	(20.71–36.11)		
EU only		50.43	(35.66–71.32)		
US and EU		34.32	(22.45–52.48)		
Single European country		27.81	(20.89–37.03)		
No. of countries involved in the trial	527	1.12^d^	(1.07–1.18)	19.58	<.0001
Year that enrolment to the protocol begins	523			4.90	0.0002
< 1990		313.70	(53.14–1852.05)		
1990–1999		26.44	(13.53–51.68)		
2000–2004		50.47	(36.25–70.28)		
2005–2009		29.75	(22.55–39.25)		
2010–2014		31.15	(23.99–40.46)		
2015 and after		35.79	(21.68–59.08)		

^a^Minimum and maximum age groups are mutually exclusive. Children, term new born infants to adolescents up to 18 years; adults, 18–64 years old; and elderly, 65 years or older

^b^Indicate whether or not a data monitoring committee (DMC) has been appointed for this study

^c^Indicate whether or not the trial includes an intervention subject to US Food and Drug Administration regulation under section 351 of the Public Health Service Act or any of the following sections of the Federal Food, Drug and Cosmetic Act: 505, 510(k), 515, 520(m), and 522

^d^Estimated coefficient, the estimated increase of sample size for every unit increase of the covariate

## Appendix 10

**Table 7:** Number of trials by conditions for females and males only and phase of study

Gender	Diseases/conditions	Phase 2	Phase 3
Females only			
	Addison Disease	1	0
	Congenital Toxoplasmosis	0	1
	Gastroschisis	0	2
	Hereditary Breast and Ovarian Cancer Syndrome	1	0
	Lymphangioleiomyomatosis	3	0
	Ondine Syndrome	0	1
	Ovarian Cancer	48	11
	Peripartum Cardiomyopathy	1	0
	Placental Insufficiency	1	0
	Preeclampsia	1	1
	Rett Syndrome	5	0
	Systemic Sclerosis	1	0
	Turner Syndrome	0	1
Males only			
	Allan-Herndon-Dudley Syndrome	2	0
	Barth Syndrome	1	0
	Becker Muscular Dystrophy	1	0
	Duchenne Muscular Dystrophy	6	2
	Fabry Disease	2	0
	Fragile X Syndrome	4	0
	Hemophilia	0	1
	Hemophilia A	1	2
	Severe Hemophilia A	1	1
	X-Linked Hypohidrotic Ectodermal Dysplasia	1	0

## Appendix 11

**Table 8:** List of rare diseases as identified by Orphadata and in AACT but with unknown class of prevalence

Diseases/conditions	No. of trials, *m*	(%)
Allergic bronchopulmonary aspergillosis	1	(2.70)
Amyloidosis	5	(13.51)
Arachnoiditis	1	(2.70)
Aspergillosis	5	(13.51)
Cutaneous mastocytosis	1	(2.70)
Erdheim-Chester disease	2	(5.40)
Fibrous dysplasia of bone	2	(5.40)
Germ cell tumor	3	(8.10)
Heparin-induced thrombocytopenia	1	(2.70)
Hereditary pulmonary alveolar proteinosis	1	(2.70)
Idiopathic nephrotic syndrome	1	(2.70)
Intrahepatic cholestasis of pregnancy	1	(2.70)
Kawasaki disease	1	(2.70)
Ligneous conjunctivitis	1	(2.70)
Loiasis	1	(2.70)
POEMS syndrome	2	(5.40)
Precocious puberty	3	(8.10)
Solitary fibrous tumor	1	(2.70)
Synovial sarcoma	1	(2.70)
Thymic carcinoma	2	(5.40)
Zygomycosis	1	(2.70)

## Abbreviations

AACT: Aggregate analysis of clinicaltrials.gov
CHMP: Committee for medicinal products for human use
CI: Confidence interval
DMC: Data monitoring committee
EMA: European medicines agency
FDA: Food and drug administration
ICD-10: 10^th^ International classification of diseases
ICMJE: International committee of medical journal editors
INSERM: French National Institute of Health and Medical Research
IQR: Interquartile range
MedDRa: Medical dictionary for regulatory activities
MeSH: Medical subject headings
NLM: National library of medicine
OMIM: Online mendelian inheritance in man
RCT: Randomised controlled trial
UMLS: United medical language system
